# The nexus between Egyptian renewable energy resources and economic growth for achieving sustainable development goals

**DOI:** 10.1186/s43093-021-00091-8

**Published:** 2021-10-10

**Authors:** Doaa Salman, Nadine Amr Hosny

**Affiliations:** 1 Head of Economics Department, Associate Dean, October University for Modern Sciences and Arts, Cairo, Egypt; 2 October University for Modern Sciences and Arts, Cairo, Egypt

**Keywords:** Sustainable development, Economic growth, Renewable resources, Egypt, Q01, Q2, O1

## Abstract

This study contributes to the conceptual and empirical studies by investigating the relation between the electricity generated from renewables, carbon dioxide (CO_2_) emission, exchange rate and unemployment on Egyptian economic growth (EEC). Developing countries are in pursuit of economic growth as it is the path for sustainable economies. The study applies autoregressive distributed lag model (ARDL) using the dataset for the period from 1990–2019. The empirical results highlight the main driving forces that accelerate economic growth. The main findings confirmed that government support is one of the key drivers for positive and significant impacts of electricity generated from renewable energy sources, CO_2_ emission, and exchange rate in Egypt on economic growth. However, the positive and significant impact of carbon dioxide still plays a challenging aspect to achieve sustainability. Policies have been identified to develop the required energy network of the future

## Introduction

Study-based modeling presents a concise trade-off relation between EG (economic growth) and SD (sustainable development). Growth built on energy is a double-sided sword—nothing is for free. To achieve a balance between EG and economic development which is a long-term target, it requires creating a balance between utilizing resources without exploitation. This balance will require the government to monitor the behavior of the economic activities and to regulate it to achieve SD and growth. However, developing countries, they stimulate investment to create growth first and later they aim for development as a second priority. Such a prioritization is the main reason for the delay of the development. The quest of the developing countries to increase growth rates and improve the well-being of people still contributes to be far objectives as it has been for the past decades. Economic growth at an increasing pace is essential to improve living conditions. Hence, reducing the economic gap through clean production can be seen as a longer-term objective. The high rate of population growth is continuing, and in the meantime, there is an urgent need for economic growth to catch up with the rest of the developing and developed world. An increase in energy generation is essential to meet the population and economic growth and support the improvements in the quality of life.

In 2015, Egypt adopted the ESDS (Egypt Sustainable Development Strategy) vision 2030. Its main objective is to achieve a competitive, balanced and diversified economy by 2030 to secure SD and to protect the environment. Thus, the Egyptian government took bold steps toward adopting an energy diversification strategy with increased development of RE and implementation of energy efficiency (IRENA 2018a). This paper focuses on how Egypt is capable of transforming the usage of nonrenewable to renewable resources or not. It explores the relation between RE and EG through ARDL (auto-regressive model) and correlates it to the sustainable development goals. In this paper, main research questions are: Will RE implementations have a significant impact on EG? What are the challenges that Egypt have faced and will face to achieve sustainability?

The proposed hypothesizes are: if Egypt will have the ability to improve and develop the application of RE as a replacement for the usage of non-RE and if this will positively affect the Egyptian economy, then SD will occur in the end.

The following sections start with section one which includes: a review of the literature by showing the significant of the different RE forms. Section two represents the model developed. Section three includes the empirical results. Finally, the last section presents the conclusion and paper recommendations given for the country to strengthen its ability to achieve SD.

## Literature review

### Prospect roles of renewable energy (RE)

RE is important part of the evolution the countries worldwide are achieving as it plays a huge role in raising the economic level and increasing the innovation. RE minimizes the cost of production and provides many alternatives for the country to choose from when they face a certain situation. There are many kinds of RE as solar, wind, geothermal, hydroelectric, hydrogen and fuel and many more types. According to (Panwar, Kaushik, Kothari, 2010), a crucial role will be played by RE in the world’s future energy resources that are divided into three types, the first one is fossil fuels and then there is renewable resources and the last type is nuclear resources. SD achieved when economic development can happen without the depletion of any of the resources. In addition, it means that the resources currently will consume without reducing the opportunity of the upcoming generations. Regarding energy usage, achieving sustainability condition can be if these types of energy reloaded, this is the first condition. Second condition is any improvements in technology will increase the ability of achieving increase in the resources. The third condition involves that these resources have long-term availability. All types of RE whether solar, wind, hydro, tidal or geothermal can be all classified as sustainable energy [14]

The requisite for RE around the globe increase as it stops the pollution caused by another sources of energy such as coal—which pollute the environment. Focusing in DC, the RE will help in improving energy supply reliability, organic fuel economy, dealing with the task of local energy and supplying water also developing the standard of living and the local population level of employment, making sure there’s a regular improvement in the distant places in the deserts and mountains zones. It will help in international agreements made to protect the environment in the future especially with problems like global warming affecting the future. Expanding and making more RE stations will help in providing more job opportunities especially in poor areas, which will decrease the immigrants from these poor places to find job opportunities in other regions. Similarly, RE is affordable, sustainable, and reliable as well as it does not pollute the environment (Panwar, Kaushik, Kothari, 2010). Later, Shahzad [17] show that RE is highly demanded now and needed as it’s an infinite supply which never ends and most of its costs are spent on people and assets which once generated its cost will drop than before. In addition, it is a new line of business in which many investors will focus in investing in it as it’s predicted to be very essential for people in the future as the fossil fuel is going to be consumed at last. However, RE is going to last and is more cheap once its basis is contracted and the infrastructure is ready.

Historically, there were many uses for RE one of which is water desalination which requires large amounts of energy for the process of separating salts from seawater. This process is extremely costly and very few Middle Eastern countries can afford this process. The water supply from desalination expected to increase tremendously in the upcoming years that may cause some challenges. One of these problems or the most essential one is related to energy supply and environmental issues like pollution that are triggered by the usage of the nonrenewable resources as fossil fuels. Around 203 million tons of oil per year is required to produce 22 million m/day with the thinking going on the environmental issues caused by the usage of fossil fuel. It was considered using oil more as it is accessible and if it was manageable to burn by the amount needed to provide the demand of water by the people but after considering the vitality of the CO_2_ levels and the greenhouse effect oil usage eliminated, Karim et al. [8]. Delgado et al. [3] stated that one of the RE sources that are used in the process is solar desalination as it is used in to produce fresh water by stimulating rain which is the number one provider of fresh water. Moreover, solar radiation makes water of the sea evaporate then the water rises, moved by the winds and cools down. Then it causes rain to happen and fresh water to be generated. Sources that are freely found in nature is used in this process making it more easy to produce fresh water and with this solution tested in any places it has shown success and that it is efficient to be used and its main focus is that it doesn’t harm the environment.

The DC should center its focus on providing and investing in the nonrenewable resources as it is cheap and can stabilize the prices as its cost is only in the invested capital and it will decrease the unemployment scale. These countries will dodge the safety problems of fossil fuel as collapsing “Coal mine” and explosions on oil platforms.

### Renewable energy and its impact on economic activity

Using wind energy is very useful as it has insignificant maintenance costs, very few waste products and it doesn’t cost much making a wind turbine and has a long-life span; however, it also has its cons such as it could cause noise pollution and damage the soil. Also, using hydro-energy to generate electricity has its edge such as protecting the land from any flood threats, it is highly efficient (calculated at 80%), minimum carbon dioxide emissions so it is environmental friendly. Finally, hydro-energy is used in a form of dams and they’re very useful as they have a long-life span, functioning for 100 and more years, Ellabban et al. [4].

The perfect substitute from RE to petroleum and fossil fuels is biofuels. There are many pros for using biofuels. Biofuels can perfectly adapt with changes in climate and its cost and prices are reasonable. Furthermore, it is environmentally friendly since it causes reduction in the greenhouse emissions. This is due to the generation of fewer toxins captivated by plants. In addition, it is produced locally in all countries and thus this reduces the cost of importing other resources. On the contrary, there are some drawbacks for using biofuels. Firstly, it requires huge land areas in order to meet the local energy demand. This can lead to destruction of living habitats. In addition to, biofuels require huge amount of capital to distill it. Roy and Das [15] show that the usage of RE affects the entire global GDP and affects unemployment rates.

Another advantage from the usage of RE is the decline in energy expenditure. If the dependence on RE increased efficiently, then the electricity bills will decline dramatically. Furthermore, the generation of RE will increase the ability of each country to be self-sufficient of energy sources. This will decrease the need to import oil from foreign countries and thus there will be trade surplus that leads to EG, Rüdiger [16]. Finally, yet importantly, setting up RE sources on lands will increase the property prices significantly, as people believe that it is more beneficial purchasing a land with solar power installed on it as the process of maintaining and installing one is quite a difficult task. An increase in property value stimulates the purchasing of the consumers and as a result, it achieves a higher EG.

## Renewable energy in the middle-east countries and Egypt

### Renewable energy in middle-east countries

First of all, the energy sector in the Middle East (ME) plays a major role in achieving economic development. Thus, it also affects the global economy as a whole. Throughout the previous couple of years, many concerns regarding renewable energy in the ME countries have been raised. This was due to the various issues raised regarding global warming and the fossil fuels depletion. Apart from that, the ME has a great potential for renewable energy implementation due to its geographical and environmental traits. This is applied specifically for the solar and wind energy [6]. Examples of countries with heavy energy consumption and high carbon dioxide emissions are the United Arab of Emirates and Saudi Arabia. Lately, these countries took huge steps regarding using and producing clean energy on a large scale in order to reduce CO_2_ emissions and achieve sustainable development [2].

Furthermore, UAE, Saudi Arabia and Qatar invested heavily with multi-billions of dollars in plans and projects to develop alternatives regarding energy sector. The most noticeable example is the Masdar city of Abu Dhabi. This city made enormous changes and approaches to fight against global warming and use latest technologies for achieving sustainable energy. This city will be zero carbon, and zero waste modern urban habitat. Also, the city promotes development in a modern clean tech cluster and free economic zone [2]. UAE especially made tremendous efforts to increase the awareness of people concerning the importance of renewable energy. Also, there are many strategies implemented to increase the capacity of renewable energy. Such strategies are: UAE Vision 2021, the UAE energy strategy 2050, The Dubai clean energy strategy 2050 and Abu Dhabi economic vision 2030. So, for example one of the goals of UAE vision 2021 is to achieve sustainable environment by raising the contribution of clean energy by 27% in 2021 compared to 0.23% in 2015 [1].

Egypt, Jordan, Morocco and Saudi Arabia set its strategies to develop clean energy program for the upcoming generations [2]. Countries aim to increase their power capacity to reduce (greenhouse gases) GHG after signing the Paris climate agreement in 2015. Iran plan to reduce its GHG emissions by 4% in 2030. Furthermore, there is cooperation between Iran and Germany regarding renewable energy application. Due to this cooperation, Iran is expected to increase the renewable potential from 3% to more than 38% in 2030. Also, it is expected to have more than 100 GW of power capacity. While, Kuwait power system depends on the hydrocarbon resources and their aim is to increase the renewable energy by 15% in 2030, [6]. The frequent increase in demand on power and energy is the future challenge especially from the countries that depend heavily on industrialization and they seek the resource with the lowest cost.

### Renewable energy in Egypt

One of the major contributors for the development in Egypt is the energy sector. It generates more than 20% of the total GDP and more than 300,000 people are employed in such sector in year 2017, Springborg [18]. The problem here is that since year 2007, the country faced many obstacles in such sector especially in the electricity due to the tremendous increase in electricity consumption. Regarding the renewable energy, from the beginning of year 2008, the awareness concerning the crucial importance of RE has increased. This was obvious by adopting new national RE strategy by Egyptian solar plan and by announcing the feed-in tariff for wind and solar photovoltaic (PV) projects. There was tax reduction applied for the renewable equipment in 2014 and offering tax incentives for RE in 2015 (Dr. Ulrike Lehr [4]). According to the Egypt vision 2030, the usage of RE and using energy efficiently will have a positive impact on the EG. If RE projects will rise and increase, then this will create more job opportunities that will definitely reduce the unemployment. The usage of RE will encourage businesses and firms to put into consideration opening businesses with the aim of providing RE. This will increase the domestic competition, and the competition among different countries will increase since the high-tech inputs needed for energy can accelerate exports. In addition, substituting energy to the usage of more RE will have a direct positive effect on subsidies. Giving more support for investments of RE to switch the usage of diesel generators to solar pumping especially in agriculture, this will give the chance for many small domestic firms to compete in such field and this will replace the subsidy for diesel.

On the contrary, the wide increase in the RE may have adverse effects on the other sectors of the economy. First, the expansion of the PV projects in order to generate electricity will require the increase for payments given to these projects and the budget left for the other macroeconomic investments will be very small. The demand for investment goods will be decreased especially in the sector of construction. It was expecting in 2020 that more than 25% of the total exports would generate from RE. Increase in exports of RE will lead to decline in other sectors, which titled “Dutch disease.” Accordingly, the current account balance will increase since exports increased thus causing currency appreciation. Accordingly, the competitiveness of the products exported will decline such as textiles, chemicals, machines, and even private services such as tourism and Suez Canal services [13]. Regarding the impacts of RE on household incomes and poverty, increase in RE investments will increase the national GDP automatically and thus this will cause the incomes of people to rise. Nevertheless, this is not applicable to all people since there are differences between various groups of households. Especially the people in rural areas will benefit from the RE contribution since the lower income group of people will receive more income as a return for labor input. In addition, the people living in urban areas will benefit especially those endowed with the high technical skills that used in RE projects such as technicians and operators.

Egypt is a fast-growing population between all countries in North Africa and the Arab region. This tremendous increase in the population led to an automatic increase in the demand for energy, which led to huge shortages in the domestic energy resources. Accordingly, in order for the country to be capable of meeting the demand for energy, the Government established an energy diversification strategy titled by ISES “Integrated Sustainable Energy Strategy” which can be achieved by 2035. The main aim of such strategy is to maintain the ability of the country to supply the amount of energy that meets the demand of people. By fulfilling this, it can increase the development of RE and increasing the efficient usage of energy [7].

**Fig. 1:**
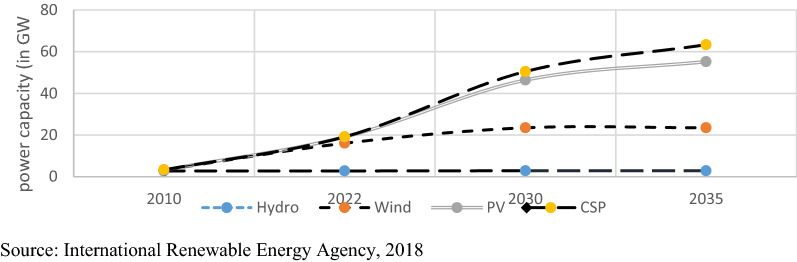
Current and potential installed capacity of renewable energy in Egypt in Gigawatts. *Source*: International Renewable Energy Agency [7]

The installed capacity of the RE in the previous and the upcoming years their main sources of RE are coming from hydropower and the rest came from solar and wind power. Generally, Egypt enjoys the existence of many sources of RE such as solar, wind, biomass and hydropower, see Fig. 1. Since 1970s, Egypt implemented and undertook many fruitful decisions regarding using technological tools for RE sources. This was in coordination with many other countries such as France, Germany, Italy, Spain and the USA. Due to this cooperation, there were obvious Egyptian improvements in the installation of solar water heaters (SWH), wind farms and photovoltaic (PV) applications in pumping water and plant desalination. The solar energy intake of the daily sunshine ranges from 9 to 11 h per day—with an estimate of solar direct radiation intensity from 2,000 to 3,000-kilowatt hours per square meter. This is an enough amount of solar energy that utilized efficiently. Apart from solar energy, the wind comes at average annual speeds of 8 to 10 m per second near the Red sea and 6 to 8 m per second near the Nile and Western desert. In addition, more than 30 million tons of biomass waste are coming from agriculture—the current problem is that these resources are unused.

**Fig. 2:**
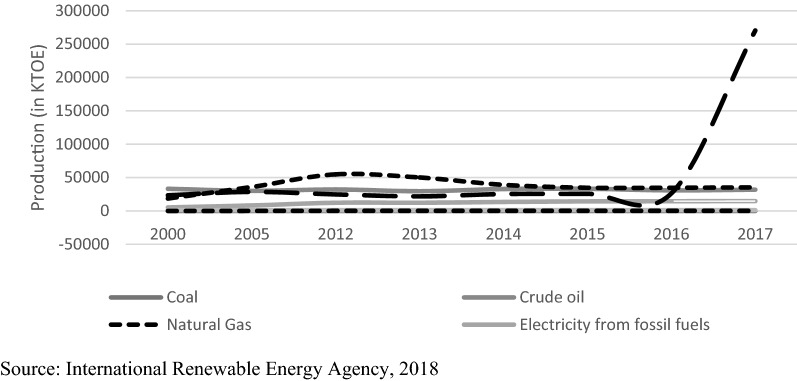
Total primary energy supply in Egypt from 2000 to 2017. *Source*: International Renewable Energy Agency [7]

In the above figure, it shows the energy production of Egypt from different sources whether renewable or nonrenewable from year 2000 to 2017. This figure shows whether it will be in line with the sustainable energy strategy implemented or not. From this Fig. 2, it is obvious that the main energy production is comprised of oil and natural gas. However, on the contrary the generation of the renewable energy is not utilized until the previous years. In 2010, the contribution of the RE was not that high since it comprised only 4% of the total energy production, which came mainly from hydropower that was almost 3%, and only 1% came from wind power. However, by 2021/2022, the contribution of the RE expected to increase to reach 8% by the end of 2035 to become 14%.

*Hydroelectric energy*: In Egypt, the main reference for the hydropower is the river Nile especially located in Aswan where there are various power stations located there amounted to 2,800 MW (Megawatts). Furthermore, these power stations help in generating electricity of an amount totaling 13,545 GW (gigawatts) annually. Around 50% of the electricity in Egypt in the 1960s and 1970s was generated from the hydropower, this is due to the rise of many thermal power stations, generating electricity from hydro-power has declined dramatically to reach 7.2% only in 2015/2016 (International Renewable Energy Agency [7]).

**Fig. 3:**
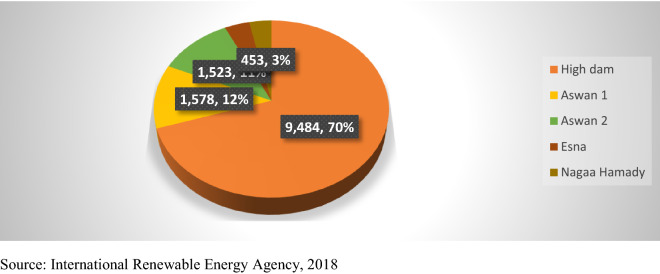
Hydroelectric stations and their capacity in year 2015/2016 (In GWH). *Source*: International Renewable Energy Agency [7]

Figure 3 depicts the five main hydroelectric stations that generate electricity per year. There was a potential to increase the hydroelectric plants by establishing another four additional power stations in “Assuit” by the end of 2018. Also, there were plans to implement a 2,400 MW (Megawatts) pumped storage hydroelectric plant in “Attaqa” by the end of 2022 (International Renewable Energy Agency [7]).

*Wind energy*: Wind, eventually determined by environmental air, is simply another method for gathering Energy. Sun likewise warms the environment, which produces wind. It takes a shot at shady days what's more, Rainy season too. The area of wind turbines is an important factor, which affects the presentation of the machine. According to Egypt’s Wind Atlas, the country is full of wind resources and considered—especially in the area of the Gulf of Suez—one of the best locations internationally for generating wind energy. Due to the availability of high stable wind speeds on average of 8 to 10 m/s at a height of 100 m. In addition, this location is characterized by the abundance of huge uninhabited areas of desert. Furthermore, there were new areas discovered east and west of the Nile in many areas such as “Beni Suef,” “Menya” and “ElKharga oasis.” These areas have an average wind speed estimated between 5 and 8 m/s and they are appropriate locations for generating electricity and for water pumping. It is expect by 2023 to have four wind plants fully established. These four plants have an estimated wind capacity of 2,610 MW. Apart from that, there are already various energy projects made by Siemens with 2,000 MW installed capacity (International Renewable Energy Agency [7]).

*Solar energy:* There are two types of solar energy that are Latent sun based and Dynamic Sunlight based. Latent sun based is the usage and utilization of the immediate energies coming from the sun. Dynamic Solar Energy is the utilization of the sun's electro-attractive radiation in producing electrical energy. The Egyptian intake of the solar radiation makes it capable of generating electricity and using solar energy for other thermal heating applications. Recently, Egypt planned to invest into building the hugest solar photovoltaic plant in the world at “Benban” which is located in Aswan. This project cost around 4 billion dollars is estimate to produce more than 1.8GW of power [9]. The main roles of such solar PV projects are pumping, lighting, advertising and desalination. After 2014, the electricity shortage that happened in 2014 along with the decline in the costs of PV panels, many Egyptian authorities has drawn efforts regarding PV applications. There are other two main solar PV plants was expected to be done by late 2019. It was planned to be mainly located in “Hurghada” and “Kom Ombo,” respectively. The first Solar PV plant financed by Japan and the other plan financed by France. It expects that these plants to reduce the CO_2_ emissions by 40,000 tons and to produce around 32 to 42 GWH annually (International Renewable Energy Agency [7]).

*Biomass:* is the most significant hotspot for vitality creations provided by farming. Biomass vitality created when natural issue changes to energy. The conversion of biomass is an environmentally friendly process and financially applicable. Furthermore, Egypt is known with the abundance of resources that can be used in biomass generation. These resources can come from agricultural wastes, urban solid wastes or animal fertilizers. First, the waste extracted from agriculture composes around 35 million tons per year. Such waste has other usages such as energy purposes. Almost 60% of the waste used for such energy uses. Regarding the urban solid waste, Cairo alone contains 10,000 tons of waste daily. Therefore, in the current years, there were many new technologies adopted and innovated for biomass such as the technologies needed for the production of biogas from the waste coming from animals especially in rural areas. These kinds of technologies created many job opportunities in such areas and thus the rate of migration of young people from rural to urban areas have declined (International Renewable Energy Agency [7]).

### Environmental performance index in Egypt

The environmental performance index (EPI) is an index that measures the environmental standards in each and every country. It created by Yale and Columbia University in coordination with the World Economic Forum and the Joint research Centre at the European commission. Therefore, the EPI index examines to what extent does this country protects its environment and to what extent there are improvements done to enhance the environmental performance. The EPI index combines the performance of many indicators including many indicators related to the ecosystem volatility and environmental health. Such indicators include health impacts, air quality, water sanitation, water resources agriculture and biodiversity (Reut institute, n.d.).

**Fig. 4:**
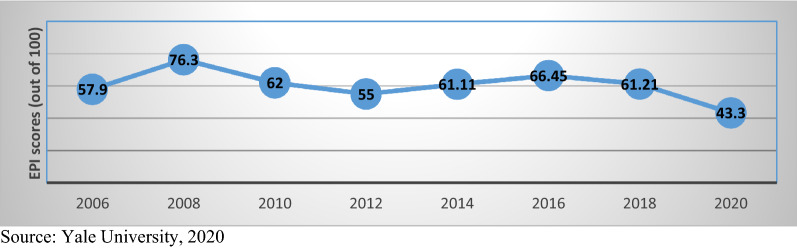
Environmental performance index (EPI) in Egypt (out of 100). Source: [24]

**Fig. 5:**
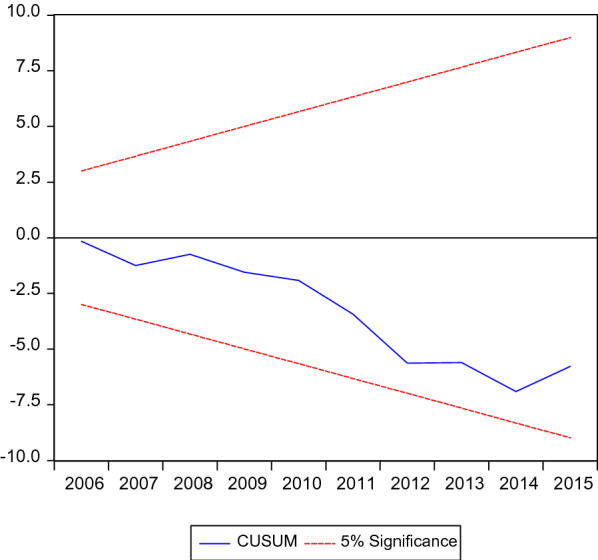
Cumulative sum of recursive residuals

Figure 4 illustrates the EPI of Egypt since 2006. Therefore, according to this index, it measures four main components as mentioned above. From this Fig. 5, it is clear that there was a huge improvement in the EPI score after 2006 especially in years 2008 and 2010. First, regarding the air quality, there was 87 stations associated to the National Network for monitoring air pollutants in Egypt widely distributed among various areas and regions in Egypt. Also, in year 2010, many cement companies collaborated with the National Network for monitoring air pollutants in order to observe the daily emissions made by these companies. Furthermore, concerning the emissions made by the vehicles, Ministry of State for environmental affairs (MSEA) agreed with the traffic and environmental police to inspect cars causing high emissions on roads [11].

**Fig. 6:**
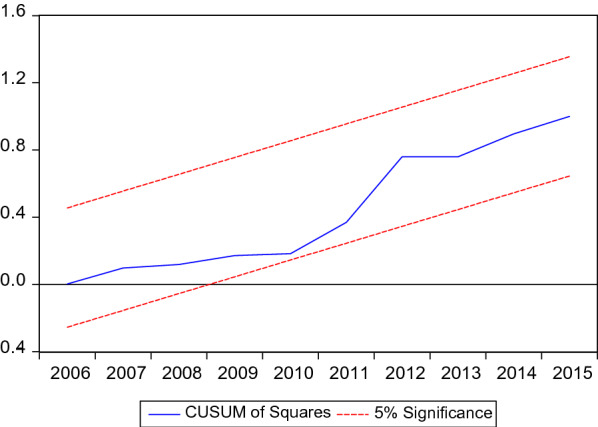
Cumulative sum of square recursive residuals

Apart from the air quality, the EPI score included the climate change as an indicator. After examining this indicator, many reports concluded that Egypt is prone to many risks and threats regarding climate change such as increase in the temperature levels increase in the sea level and shortage in the water resources. These risks will have negative impacts on the public health and infrastructure. Therefore, as a result, the country aimed to collaborate with the UNFCCC to reduce the GHG (greenhouse gas emissions). More than two projects expected implementation in order to reduce the carbon dioxide emissions by 9 million tons. Such projects will cost approximately 3 billion pounds [11]. After 2010, the EPI score of Egypt declined slightly compared to other countries globally but then in began to increase again after 2014. In 2018, Egypt as a rank jumped 40 points from being the 104th country to become 66th country. This gives an indication of the tremendous improvements that were taken concerning saving the environment in the previous years. This was mainly due to the increasing concerns made by both the public and private sector regarding adopting environmentally sustainable solutions. For example, the application of electric cars and buses and the non-profit organization of “Bassita” collaborated with Greenish to clean all the wastes found in the Nile and the shores. Unfortunately, due to the corona pandemic and due to the downturn of the economy in Egypt in 2020, the country was not able to increase its concern regarding environment but it declined further to reach 43.3 [5].

### Egypt and sustainable development goals

SDGS goals include eradicating poverty in all of its forms across the globe—which by 2030 intent to be achieve. As well, hunger termination, offering sufficient food supply and promoting well-being. Technically, Egypt is heading toward achieving SD goals since the SD strategy (Egyptian Vision 2030) is in line with SDGs. Lately, Egypt has depicted an obvious improvement in the infrastructure across various sectors. Also, the energy sector perceived a huge increase in the available capacity whether to produce, transport or distribute electricity. Currently electricity produces at least 15 gigawatts and the amount of RE usage increases by more than 42% by the end of 2035. Lately, the government has enhanced the opportunities for private sector firms to enter the electricity field [19].

### Challenges for renewable energy in Egypt

Egypt strategy aims to be fulfilling by 2035 that includes maintaining technical and financial stability for energy sector in specific through RE application. In order for this action to be applicable, this requires from the government to try to resolve all the obstacles that might hinder the development of RE. One of the obstacles that the government might try to put into consideration is providing the latest and the most advanced technologies for RE development. The government must consider monitoring the prices of resources. This is necessary in order to reduce the cost used for RE. Added challenge that might postpone the rapid expansion of the RE application in Egypt is the lack of infrastructure. The infrastructure needs to be more advanced since this lack causes a delay in the expansion of the rooftop PV installations. Accordingly, this limits the market growth in Egypt since most of the rooftops are misused in the country due to the existence of satellite dishes and so on. The rules and regulations that adopted until date are concerned more with the usage of wind and solar energy to generate electricity, but on the other hand, there was not any focus toward the potential of biomass. In addition, there is not any financial support given to the biomass. This is mainly due to the high cost of using biomass as a source for electricity generation.

There are many efforts done by the government to promote the usage of RE and to encourage the private investors regarding the RE application. On the contrary, many of those private investors faced many obstacles including the lack of existence in the contracts for these types of projects. In addition, the existence of fuel subsidies will create market distortions since this impedes the expansion of the RE and prohibits the ability of the RE to compete on the same price level with fuel. Furthermore, the application of the PV systems to generate electricity was costly due to the current exchange rate. The current exchange rate regime dampens the possibility of any international funding opportunities. In addition, the commercial interest rates on loans given to the SMEs (small medium enterprises) are almost 18%. This interest rate considered very high; it actually discourages the SMEs from trying to invest in the RE sector (International Renewable Energy Agency [7]).

## Methods

The general frame work is explicitly designed for the purpose of analyzing the macroeconomic and sectorial effects of environmental policies on economic growth. The study applies that the ARDL cointegration technique to examine the impact of using RE on GDP growth (EG) is as follows:

$${\text{GDP}}\;{\text{ growth}}\;{\text{rate}} = \alpha + \beta_{1} \;{\text{Electr}}.\;{\text{ from}}\;{\text{renewables}}\left( {\text{\% }} \right) + \beta_{2} \;{\text{Unemp}}.\;{\text{rate}} + \beta_{3} \,{\text{Exch}}.\;{\text{rate}} + \beta_{4} \;{\text{Co}}_{2} \;{\text{emissions}}$$

The dependent variable: GDP growth rate (%). The independent variables are: electricity generated from renewables (%), inflation rate (%), unemployment rate (%), CO_2_ emissions (metric tons per capita) and exchange rate (LCU per $ US, period average). The data used in this paper will be from Egypt in years 1990 until 2019, World Bank [20–23].

The descriptive data analysis presented in Table 1 show that economic growth and CO_2_ emission mirrors normal negative skewness while the rest of the variables are positive skewness. And all the variables are ranging between 1.5 and 2.38 < 3 that represent a Platykurtic—that means flatted curves—measure the peakness or flatten of the distribution of the series. Jarque–Bera shows that all the variables are normal distributed. In Table 2, the stationarity test for the dataset to select the suitable model to test for the model variables significance. This test examines the change of each variable over time; this can explain through unit root test. The stationary of each of the independent and the dependent variable, first, concerning the electricity generated from renewables will be better to take the first difference rather than taking it as level in order for the variable to be significant. If the first difference will be implemented, the electricity will be significant at 1%, which is the best solution, see Table 3. Therefore, in this case, we do not reject H0 and stationarity exists. Regarding, unemployment rate, exchange rate and CO_2_ emissions which are the independent variables, it will also be better to take the first difference of the data since it will be significant and we do not reject the null hypothesis. Concerning the dependent variable, which is the GDP growth rate (%), it will be significant at the first difference also and therefore the null hypothesis is accepted.

**Table 1:** Summary of the descriptive statistics (1990–2019)

	EG (%)	Exch- R	CO_2_- EMS	Elect—REs	Unemp
Mean	4.358365	4.733462	2.000692	15.63692	10.18912
Median	4.472300	4.965000	2.033150	14.35540	10.01000
Maximum	7.156300	7.690000	2.572000	23.50440	13.15400
Minimum	1.125400	1.550000	1.345700	8.259300	7.950000
Std. Dev	1.683578	1.547312	0.442337	5.387654	1.641456
Skewness	− 0.087421	0.030839	-0.139258	0.047119	0.481805
Kurtosis	2.125860	2.047867	1.525201	1.388625	2.053723
Jarque–Bera	0.860916	0.986226	2.440318	2.822527	1.899982
Probability	0.650211	0.610722	0.295183	0.243835	0.386745
Sum	113.3175	123.0700	52.01800	406.5599	254.7280
Sum Sq. Dev	70.86090	59.85439	4.891545	725.6704	64.66510

**Table 2:** Stationarity test

	Intercept-level	Trend & intercept-level	None-level	1st difference-intercept	1st difference-trend & intercept	1st difference- none
Elect—REs	ADF: 0.896	ADF:0.263	ADF:0.0031***	ADF:0.000***	ADF:0.000***	ADF:0.0173**
	PP: 0.8499	PP:0.2502	PP:0.0044***	PP:0.0000***	PP:0.000***	PP:0.0000***
UNEMP (%)	ADF:0.6687	ADF:0.0422**	ADF:0.8189	ADF:0.0059***	ADF:0.0207**	ADF:0.0002***
	PP:0.5198	PP:0.5802	PP:0.8101	PP:0.0030***	PP:0.0150**	PP:0.0002***
EXCHR	ADF:0.7409	ADF:0.1548	ADF:0.9912	ADF:0.001***	ADF:0.0056***	ADF:0.0001***
	PP: 0.6980	PP:0.2225	PP:0.9851	PP:0.0012***	PP:0.0062***	PP:0.0002***
CO2_EMS	ADF: 0.6681	ADF:0.4433	ADF:0.9542	ADF:0.000***	ADF:0.0001***	ADF:0.000***
	PP:0.6670	PP:0.4164	PP:0.9751	PP:0.0000***	PP:0.0001***	PP:0.0000***
EG (%)	ADF:0.0057***	ADF:0.0608*	ADF:0.2435	ADF:0.000***	ADF:0.000***	ADF:0.000***
	PP:0.0392**	PP:0.1432	PP:0.2598	PP:0.000***	PP:0.000***	PP:0.000***

(*), (**), (***): Null hypothesis is rejected, respectively, at 1%, 5% and 10%

Source: Authors calculation

**Table 3:** Optimal lag length

Lag	LogL	LR	FPE	AIC	SC	HQ
0	− 181.0329	NA	15.22609	16.91208	17.16005	16.97050
1	− 116.0008	94.59222	0.429221	13.27280	14.76058	13.62327
2	− 89.45032	26.55043	0.568658	13.13185	15.85945	13.77439
3	4.285145	51.12844*	0.004749*	6.883169*	10.85060*	7.817775*

^*^ indicates lag order selected by the criterion

LR: sequential-modified LR test statistic (each test at 5% level), FPE: Final prediction error, AIC: Akaike information criterion, SC: Schwarz information criterion, HQ: Hannan–Quinn information criterion

Next step is to determine the lag length for the dynamic model ARDL a VAR lag-order selection criteria, results are shown in Table 3, and it determines that the best lag length is three. Next step is employing a cointegration analysis. To determine whether the regression is spurious, results present a long-run relationship between the variables at 5% significant level, see Table 4.

**Table 4:** Johannes cointegration tests

Rank		Trace	0.05	
No. of CE(s)	Eigenvalue	Statistic	Critical Value	Prob.**
0	0.923902	136.4874	83.93712	0.0000
1	0.762490	77.24569	60.06141	0.0009
2	0.596038	44.18215	40.17493	0.0188
3	0.430893	23.33418*	24.27596	0.0654
4	0.362159	10.36939	12.32090	0.1039
5	0.001176	0.027074	4.129906	0.8930

^*^Denotes rejection of the hypothesis at the 0.05 level

^**^MacKinnon–Haug–Michelis (1999) p-values

We conclude from Table 5, that there is a long-run relationship for the model. In fact, the F-statistic is 10.541194 we compare to the Pesaran [12] critical value of 5%. According to the unrestricted intercept and trend Pesaran table, the lower-bound is 3.74 and the upper-bound is 5.06. The, F-statistic is higher than the upper-bound (10.541 > 5.85) we reject the null hypotheses. As a conclusion, there is a long relationship. Results imply that trace value tests and maximum Eigen value test we have reject null hypothesis and there is one long-run relationships among the variables. Johansen cointegration test shows that the trace statistics 23.3341 < 24.275 critical values at 5% level. Table 5 presents the existence of positive and significant relationship between economic growth and carbon dioxide emission, electricity generated from renewable energy and exchange rate. But there is a negative relation between economic growth and unemployment which supports the literature.

**Table 5:** ARDL bound test

Test statistic	Value	Lower critical bound1% I(0)	Upper critical bound1% I(1)	Lower critical bound5% I(0)	Upper critical bound5% I(1)	Cointegration
F-statistic	10.54194	3.74	5.06	2.86	4.01	yes

To determine the existing of short- and long-run relation is applied.

$$\Delta {\text{EG}}_{t} = \, \alpha + \beta_{1} \Delta {\text{CO}}_{2} {\text{EM}}_{t - 1} + \beta_{2} \Delta {\text{Unemp}}_{t - 1} + \, \beta_{3} {\text{Elect}}_{t - 1 \, + } \beta_{4} {\text{EXCHR}}_{t - 1}$$

## Results

To examine the long-run and causal relations between the model variables in Egypt, we conduct unit root tests; second, we select lag length for our variables, third, we conduct Johansen and ARDL approaches.

The findings show that all variable are positive significant at 10% except electricity for renewable energy and carbon dioxide emission at 5%. Only unemployment is negative significant with economic growth that support literature. Regarding the coefficients, it is obvious that increases by 1% in electricity for renewable energy economic growth increase by 0.7%. The increase in carbon dioxide by 1% will increase economic growth b 5.7%, see Tables 6, 7. This result shows that the impact of carbon dioxide emission is more than electricity from renewable energy from economic growth. The results support that developing renewable energy sources is essential to enhance economic growth and achieve sustainable development goal (Ibrahiem 2015; Alola et al. 2020a, b). The coefficient of unemployment rate shows that when unemployment decreases by 1% then economic growth increases by 0.4%. Finally yet importantly, once the exchange rate increases by 1%—which means depreciated—then the economic growth will increase 1.49%. The applying renewable energy enhances the economy by creating jobs in society where about 11 million jobs make globally in 2018. This creates increase in spending and income, in addition to the trend toward green growth and achieving the sustainable development goals (REN21 2019). Khobai et al. [10] find that renewable energy and investment increase the level of employment, financial development has an adverse effect on employment level.

**Table 6:** Short-run results of ARDL model

Variable	Coefficient	SE	*t*-statistic	Prob.*
ELE(-1)	0.398004	0.186399	2.135220	0.0585
EXCHR(-1)	0.837427	0.676862	1.237219	0.2443
UNEM	− 1.206881	0.297150	− 4.061525	0.0023
ECM(-1)	− 0.354338	0.246109	− 1.439759	0.0180

**Table 7:** Long-run results of ARDL

Variable	Coefficient	SE	*t*-statistic	Prob
ELE	0.786387	0.209169	3.759580	0.0019
EXCHR	1.493653	0.599297	2.492344	0.0249
UNEM	− 0.453477	0.189555	− 2.392319	0.0303
CO_2_EM	5.708392	1.577472	3.618695	0.0025
C	− 21.79266	8.025165	− 2.715540	0.0160

The long-run relation is expressed as follows:

$${\text{EG}} = 0.7864*{\text{ELE}} + 1.4937*{\text{EXCHR}} - 0.4535*{\text{UNEM}} + 5.7084*{\text{CO}}_{2} {\text{EM}} - 21.7927$$

Moreover, cumulative sum of recursive residuals and cumulative sum of squares of recursive residuals in Fig. 6 confirm the stability of the model.

## Discussion

The main motivator behind this paper was to assess the government investment in renewable energy on economic growth and to investigate the challenges that delay economic growth other than the current corona virus. The model applied dynamically showed a positive and significant impact of renewable energy on economic growth but with a negative impact on unemployment, given that with the increase of using renewable energy unemployment decrease and also with the rise of green jobs that absorb labor force, unemployment starts to fall. The usage of RE is a double-sided sword meaning that the adoption of the various strategies that have been put into consideration to be applied in Egypt in the upcoming years needs to be dealt with carefully. This is due to the extreme high costs of applying RE expansions in Egypt and the inability of the country until now to use the latest technologies necessary for the RE application. Therefore, the Egyptian government needs to be more concerned regarding the uses of RE. And the government needs to look for various ways for improving such sector.

The main obstacle that the country might face is in maintaining the economic stability while achieving SD. This is due to the highly crucial need for huge budget for expanding the RE application to meet the targets discussed in the 2030 strategy. Accordingly, this might lead to huge budget deficits at a time that the country needs to achieve EG and stabilization. In addition, the current economic situation now hinders the country’s ability to attract any foreign investments concerning the expansions in RE projects. Therefore, the question in this paper was whether the RE sources in Egypt would sustain our development or not? The answer will be “it depends,” if the Egyptian government was able to implement the goals of such strategies, then the country will be able to use RE efficiently. Nevertheless, the government needs examine ways of achieving this without harming the country’s economic wise. On the other hand, if the country mainly focused more toward achieving EG and attracting investments with these investments and expansions resulting in the depletion of its resources, then the answer for this question will be no, since Egypt will not be able to sustain the development using the resources available.

The second question that was considered and tackled throughout the paper was “will the usage of RE in Egypt strongly affect the economy?” To be able to assess this question, the paper included a regression model that was created to test the impact of RE on EG thus illustrating the effect of electricity generated from renewables on GDP growth rate. Therefore, based on data and based on this model, it was obvious that generating electricity from RE has a strong impact on the economy. Concerning whether Egypt is in line with achieving SDGS or not, the answer will be obviously yes. According to [19], Egypt has been heading toward implementing and developing the usage of RE between all sectors in the previous couple of years.

## Conclusion

To overcome the challenges that the country might face while developing the RE projects, the researchers have put forward some recommendations that might support and facilitate the process of expansion in such field. Concerning the Integrated Sustainable Energy Strategy (ISES), which expected to be applicable by 2035, it is required to involve all the essential needs concerning technological advancements and to also assess all the costs of the imported goods or resources. This strategy needs to take into account the importance of biomass energy and promote investments for such type of energy. Furthermore, the strategy needs to reconsider the importance of coal and nuclear energy in the energy supply and review their costs compared to the renewables. In addition to that, the country needs to ease and facilitate the process of paperwork of RE generation to promote the investments in such field.

Furthermore, the government needs to establish a regulatory framework regarding collecting the waste, managing it, and considering which sites should the waste be recycled and treated. Regarding the solar energy, there is an essential need to establish various ways to speed up the penetration of solar thermal systems in both the residential and industrial sectors. These ways may include obliging the real estate developers to provide each and every new building with solar thermal system. In addition, there may be a possibility of merging all the small RE projects into one large project, thus leading to achieving economies of scale. This will definitely help reduce the overall costs and thus the benefits gained by the community will increase. In addition, it will help gain more financial benefits from many investors. The Egyptian government also needs to invest in many sites and areas that might be suitable for RE projects and electricity generation. Regarding the job opportunities, there must be collaboration between RE project investors and the Ministry of Trade and Industry to enhance job creation in such sector. In addition, the investors in such a sector need a fiscal and financial incentive. Furthermore, providing the working people in such field with adequate training to improve their skills is from the top priority. Finally, such projects have to ensure the highest quality for the RE and maintain its efficiency.

## Data Availability

The data used for this research sourced from the world development indicator—World Bank.

## Abbreviations

ARDL: Autoregressive distributed lag
CO_2_: Carbon dioxide
DC: Developing countries
EG: Economic growth
ESDS: Egypt sustainable development strategy
ESDG: Egypt sustainable development goals
EPI: Environmental performance index
ISES: Integrated sustainable energy strategy
PV: Photovoltaic
GDP: Gross domestic product
GW: Gigawatts
ME: Middle East
MW: Megawatts
MOP: Ministry of planning
MSEA: Ministry of State for environmental affairs
SD: Sustainable development
SWH: Solar water heaters
UN: United Nations
UNPFCC: United Nations Framework Convention on Climate Change
